# Protein Intake and Diet Quality Mediate the Relationship Between Sleep and Handgrip Strength in Adults in the HANDLS Study

**DOI:** 10.3390/nu17111900

**Published:** 2025-05-31

**Authors:** Marie Fanelli Kuczmarski, Elizabeth Orsega-Smith, May A. Beydoun, Michele K. Evans, Alan B. Zonderman

**Affiliations:** 1 Department of Health Behavior and Nutrition Sciences, University of Delaware, Newark, DE 19711, USA; eosmith@udel.edu; 2 Laboratory of Epidemiology and Population Sciences, National Institute on Aging, National Institutes of Health, Baltimore, MD 21224, USA; baydounm@mail.nih.gov (M.A.B.); evansm@grc.nia.nih.gov (M.K.E.); zondermana@gmail.com (A.B.Z.)

**Keywords:** sleep, diet quality, protein, handgrip, healthy eating index

## Abstract

**Objective:** The aim of this study is to determine if protein intake, diet quality, or engagement in physical activity mediate the relationship between sleep quality or duration and handgrip strength. **Methods:** The sample consisted of 2171 middle-aged persons examined in the 2013–2017 Healthy Aging in Neighborhoods of Diversity across the Life Span (HANDLS) prospective cohort study. Those with sleep apnea (*n* = 222) and missing data were excluded, resulting in an analytical sample of 1308. Handgrip strength, an objectively measured variable, was determined using a Jamar Hydraulic Hand Dynamometer and expressed relative to body mass index (based on measured height and weight). Sleep quality and duration were measured using the Pittsburgh Sleep Quality Index questionnaire. Protein intake was calculated from two 24 h recalls collected using the USDA Automated Multiple-Pass Method and expressed as g per kg of body weight. Diet quality was assessed using the Healthy Eating Index (HEI) and the energy-adjusted Dietary Inflammatory Index (e-DII). Physical activity was self-reported and expressed as meeting the Life Simple 7 criterion (≥150 min/week, 0–149 min/week, 0 min/week). Mediation analysis was conducted using the Hayes PROCESS macro, model #4, for SPSS Version 4.2. Adjustment for the self-reported covariates of age (years); sex at birth (male, female); race (African American, White); poverty status (<125% or >125% US HHS Poverty Guidelines); current cigarette smoker (yes, no); marijuana, opiate, and/or cocaine user (yes, no); medical conditions including diabetes, hypertension, and/or metabolic syndrome (yes, no); and mean energy (kcal, only protein model) was performed. **Results:** Protein intake, expressed as g per kg of body weight, mediated the relationship between sleep quality and sleep duration and handgrip strength (indirect effect = −0.0017 ± 0.0006, CI 95% (−0.0030, −0.0006, *p* < 0.05); indirect effect = 0.0057 ± 0.0019, CI 95% (0.0023, 0.0098, *p* < 0.05, respectively)). Diet quality, as measured using the HEI, mediated the relationship between sleep duration and handgrip strength (indirect effect = 0.0013 ± 0.0007, CI 95% (0.0001, 0.0030, *p* < 0.05). **Conclusions:** Protein intake and a healthy diet mediate the relationship between sleep and handgrip strength, suggesting that these factors may play a role in preserving muscle strength.

## 1. Introduction

Sleep is essential for health and well-being across the life span. Although individual sleep needs vary, the American Academy of Sleep Medicine and Sleep Research Society recommends that adults regularly get 7 or more hours of sleep per night to promote optimal health [1]. Yet 28.3% of men and 27.2% of women aged ≥ 18 years residing in the United States (US) sleep less than 7 h on average within a 24 h period [2]. Sleep duration and quality can impact other lifestyle behaviors, such as food choices and physical activity, which can affect the risk of developing chronic conditions [3]. The relationship between sleep duration and handgrip strength, an important biomarker of an individual’s health [4], has been documented in multiple populations [5,6,7]. Greater handgrip strength has been associated with better sleep quality [8,9].

Research has been conducted on the direct associations between diet quality and sleep, as well as between diet quality and protein, on handgrip strength. In addition, there are publications that explore the direct associations between physical activity and sleep, as well as between physical activity and handgrip strength. The exploration of multiple lifestyle behaviors in one conceptual model is limited. Mediation models allow for the examination of the relationships between the dependent variable, sleep, with the independent variable, handgrip strength; the relationship between sleep and lifestyle behavior(s), the mediator(s); and the relationship between handgrip strength and lifestyle behavior(s). There is a paucity of information on the role of diet and physical activity as parallel mediators in the relationship between sleep and handgrip strength. Diet-related factors can include overall diet quality and/or the intake of a specific nutrient, like protein intake. This study’s objective was to determine if the relationships between sleep quality or sleep duration and handgrip strength, adjusted relative to BMI, is mediated by diet quality, protein intake, and/or physical activity. Given the consistent and strong evidence of the positive associations between diet and sleep and between diet and handgrip strength, we hypothesize that protein intake and overall diet quality will have direct and indirect effects on the sleep–handgrip strength relationship. Based on our current knowledge of the impact of activity on sleep and handgrip strength, we hypothesize that activity will have direct and possibly indirect effects.

### 1.1. Background of Sleep, Handgrip Strength, and Diet

There is a bidirectional relationship between diet and sleep [10,11], such that better sleep is associated with higher diet quality [12]. Studies have revealed that sleep duration is associated with diet quality [13] and protein intake [14], while sleep quality is associated with diet quality [15] and eating behaviors [16]. Low-quality diets characterized by high total energy and fat intake and low fruit intake have been associated with short sleep duration [13,17]. Furthermore, greater consumption of sugar-sweetened beverages has been linked to shorter sleep duration in adults and children [18], while habitual protein intake has been positively correlated with sleep duration. Importantly, persons described as normal sleepers consumed more protein than did people with insomnia [14], and diets generally rich in fiber, fruits, vegetables, and anti-inflammatory nutrients and lower in saturated fat have been associated with better sleep quality [10,15].

Relationships also exist among protein intake, diet quality, and handgrip strength. Our previous research found that high diet quality, along with total protein and essential amino acid intakes, were associated with better handgrip strength relative to body mass index (BMI) [19,20]. Other researchers have also reported on this relationship [21,22,23]. Jun and colleagues documented that a dietary protein intake of ≥1.2 g per kg of body weight was associated with greater handgrip strength relative to BMI, compared to a lower protein intake, among middle-aged adults in the 2011–2014 National Health and Nutrition Examination Survey (NHANES) [23]. Across all US adult participants in the NHANES, 2011–2014, handgrip strength increased with increasing quartiles of dietary intakes of total protein, animal protein, plant protein, and leucine [21]. Using data from the Korean National Health and Nutrition Survey, 2016–2019, Kim and colleagues reported that older women with protein intakes higher than the estimated requirement were at lower risk of low handgrip strength [22]. To our knowledge, there are no publications exploring the role of protein or diet quality as mediators of the relationship between sleep and handgrip strength.

### 1.2. Background on Sleep, Handgrip Strength, and Activity

Physical activity’s or energy expenditure’s relationships with sleep remain unclear in comparison with those between energy intake and sleep. The findings of a systematic review of the literature revealed that moderate physical activity was positively associated with sleep quality and enhanced sleep duration. However, vigorous physical activity resulted in difficulties falling asleep and poor sleep quality [24]. Another review found that the associations of physical activity with sleep quality, sleep efficiency, and waking after sleep onset were inconsistent [25]. The relationship may be bidirectional, but overall the literature is mixed regarding the direction of this relationship [25]. It does appear that daily fluctuations in sleep impact physical activity, specifically that sleep activity predicts a moderate next-day level of physical activity in healthy young adults [26] as well as in older adults [27].

The relationship between physical activity and handgrip strength, like sleep quality, is unclear. In some cases, physical activity was not related to handgrip strength [28]. It is widely recognized that handgrip strength declines with age, probably due to reduced activity [29]. Handgrip strength is a critical factor for healthy aging [30,31]. A review of the literature revealed exercise training can potentially improve handgrip strength in older adults [32,33]. The change seems to be dependent on the mode, duration, and intensity of the exercise. Task-specific exercise training or activities that focus on hand strength or incorporate handgrip strength may lead to improvements in handgrip strength [32]. It is possible that strength-building physical activity plays a mediating role in the relationship between sleep and handgrip strength.

## 2. Methods

### 2.1. Sample

**Figure 1:**
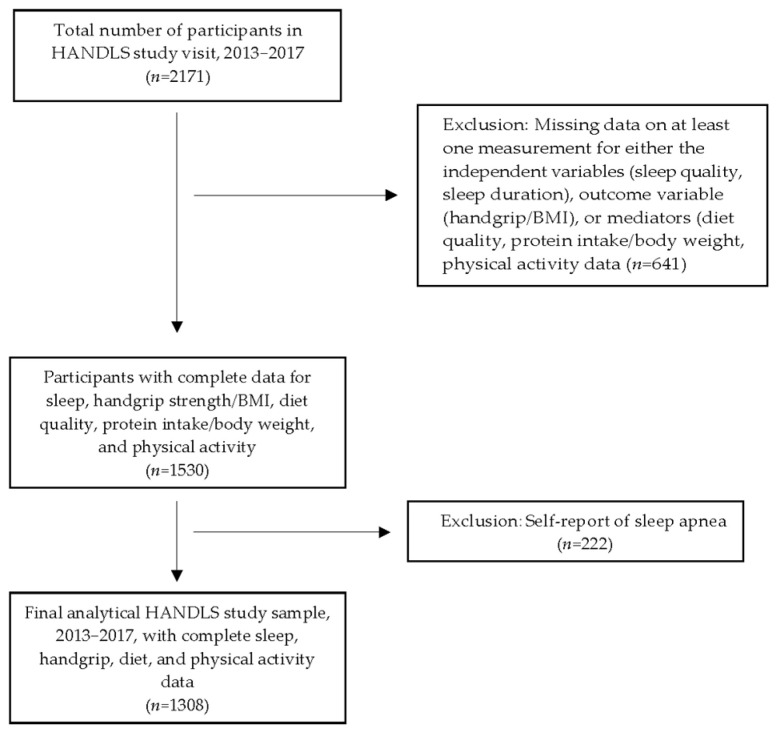
Participant flowchart. Abbreviation: HANDLS, Healthy Aging in Neighborhoods of Diversity across the Life Span.

The sample consisted of 1308 individuals with complete data on independent, outcome, and mediator variables who were interviewed and examined in the second follow-up wave of the HANDLS study, 2013–2017 (*n* = 2171) (Figure 1). Participants missing data on any of the independent, outcome, or mediator variables or self-reporting sleep apnea were excluded from the analytical sample (Figure 1). The review board of the National Institutes of Health approved the protocol and written informed consent was obtained from all Healthy Aging in Neighborhoods of Diversity across the Life Span (HANDLS) study participants. This prospective cohort study was designed to explore the effects of race and socioeconomic factors on health disparities. Details of the objectives and design of the HANDLS study, a prospective study initiated in 2004, have been described in previous publications [19,34] and on the study’s website (https://handls.nih.gov/01Design.htm, accessed on 28 May 2025) The characteristics of the sample are presented in Table 1.

### 2.2. Predictor Variable: Sleep Quality and Duration

The total sleep quality score was based on self-reported responses to 19 questions inclusive of all 7 components of the Pittsburgh Sleep Quality Index (PSQI) [35]. The 7 components include subjective sleep quality, sleep latency, sleep duration, habitual sleep efficiency, sleep disturbances, and the use of sleeping medications. For each component, there was a scoring scale ranging from 0 (no difficulty) to 3 (severe difficulty). The sum of these scores, which represented the global PSQI score, ranging from 0 to 21, was used as a predictor variable. A global PSQI score over 5 indicates poor sleep relative to clinical and laboratory measures. Higher global PSQI scores indicate the worst sleep quality. Sleep duration was determined from participant responses to the question “How many hours of actual sleep do you get at night?”.

### 2.3. Dietary Measures: Quality and Protein Intake

Two measures of diet quality, namely the Healthy Eating Index (HEI)-2010 and the energy-adjusted Dietary Inflammatory Index (e-DII) were used in the analyses. These measures were selected since they reflect different aspects of the diet [36]. Both diet quality measures were based on dietary recalls collected on two nonconsecutive days. The e-DII was based on 34 of the original 45 DII components; namely, alcohol, protein, carbohydrates, dietary fiber, total fat, saturated fat, monounsaturated fat, polyunsaturated fat, omega-3 fatty acids, omega-6 fatty acids, cholesterol, 11 vitamins (A, B_6_, B_12_, β-carotene, C, D, E, folic acid, niacin, riboflavin, and thiamin), 4 minerals (iron, magnesium, selenium, and zinc), 6 flavonoid classes (flavan-3-ol, flavones, flavonols, flavanones, anthocyanidins, and isoflavones), caffeine, and tea [37]. The original DII was adjusted for energy intake using the nutrient density method [38]. The e-DII provides a measure of the inflammatory potential of the diet with lower and higher scores indicating anti-inflammatory and proinflammatory potential, respectively.

The HEI-2010 is a valid and reliable diet quality measure of conformance to the 2010 *Dietary Guidelines of America* (*DGA*) [39]. The HEI-2010 consists of nonepisodically (foods consumed almost every day by almost everyone) and episodically consumed components. The 7 nonepisodic components are total vegetables, dairy, total protein foods, fatty acids, refined grains, sodium, and empty calories. The 5 episodic components are total fruit, whole fruit, greens and beans, whole grains, and seafood and plant proteins. The maximum score of the 12 components varies between 5 and 10, resulting in total scores ranging from 0 to 100 [39]. The calculation of the HEI scores has been reported in detail in a previous publication [19].

Mean protein intake expressed in g per kg body weight was another dietary measure. Mean protein intake was also based on two 24 h dietary recalls collected by trained interviewers using the Automated Multiple-Pass Method developed and validated by the US Department of Agriculture (USDA) [40]. Codes from the USDA Food and Nutrient Database for Dietary Studies, 2013–2014, were associated with the foods and beverages reported during the recalls by HANDLS study participants [41]. This database was used to determine energy and nutrient intakes. A MedWeigh calibrated digital scale, model 2500, was used to measure body weight.

### 2.4. Physical Activity

Physical activity, specifically the time spent on moderate and vigorous activities per week, was self-reported by HANDLS study participants using the validated Baecke questionnaire [42]. Details describing the measure of physical activity have been reported elsewhere [43]. Responses to the questions on the types and amount of time spent on moderate and vigorous activities from the Baecke questionnaire were transformed into categories based on the Life Simple 7 physical activity goals [44]. The variable consisted of 3 physical activity categories: ≥150 min of moderate or vigorous activity per week (meeting the physical activity recommendations), <150 min activity (some physical activity), or no activity.

### 2.5. Covariates

Seven covariates were used in all of the PROCESS regression models. They were age (years); sex at birth (male and female); self-reported race (African American and White), poverty status (<125% or >125% of the 2004 U.S. Health and Human Services Poverty Guidelines, assigned at baseline enrollment [45]); current cigarette smoker (yes or no); marijuana, opiate, and/or cocaine user (yes or no); and medical conditions of diabetes, hypertension, and/or metabolic syndrome (yes or no). These medical conditions were selected since they are significantly associated with lower handgrip strength [46,47]. In addition, mean energy was used as a covariate in the models using protein intake.

### 2.6. Outcome Variable: Handgrip Strength

To determine handgrip strength, the maximal force in kilograms on both hands was measured using the Jamar Hydraulic Hand Dynamometer (Patterson Medical Holdings Inc., Bolingbrook, IL, USA). The HANDLS study participants were seated with their elbow resting on a table at approximately 160°. A 15–20 s rest occurred between trials. Measures were not obtained for those participants who reported pain and/or arthritis in their hand, or having hand surgery within the past 3 months that would impede their ability to successfully complete the handgrip test. Only one participant reported having surgery and was excluded from the analytical sample. For this study, handgrip strength was the mean of two trials using the dominant hand of the participant and was recorded in kilograms. For individuals who were ambidextrous the right-hand measure was used. Handgrip strength was expressed as HG/body mass index (BMI). BMI, kg/m^2^, was based on measured height and weight. Standing height was measured with the Novel Products, Inc height meter.

### 2.7. Statistical Analysis

This study is a cross-sectional analysis of data from the second follow-up visit of the HANDLS study. All statistical analyses were performed with IBM SPSS Statistics for Windows Version 29.0 (2023; IBM Corporation, Armonk, NY, USA). Means and standard errors for continuous variables and the proportion of participants for relevant categorical variables were calculated. Analysis of variance (ANOVA) and the Pearson chi-square test were performed to compare the characteristics of persons categorized by sleep quality (good vs. poor), as determined by their PSQI score. To examine the mediating roles of diet quality and physical activity on the association of sleep with HG/BMI, the PROCESS macro, model #4, for SPSS Version 4.2 by Andrew F. Hayes was used [48,49]. Model #4 was selected since it calculates a parallel mediation when the indirect effect is mediated by more than one factor. The input requirements of the PROCESS macro include a dependent variable, an independent variable, a mediator (s), and covariates. The output includes regression coefficients; significance tests; indirect, direct, and total effect estimates; and interaction terms, bootstrapped confidence intervals, and visualizations.

**Figure 2:**
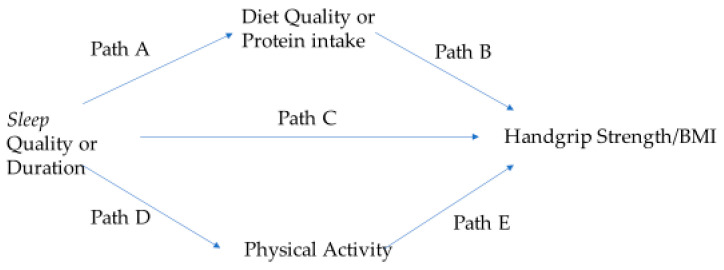
The conceptual model for exploring mediators in the association of sleep with handgrip strength. Paths A–E in the model represent direct effects. Diet quality or protein intake and physical activity are parallel mediators.

Prior to performing the Hayes Process Model #4, the 138 individuals with sleep apnea were excluded from the sample with complete data, resulting in an analytical sample of 1308. The conceptual model for the mediation analysis for this study is displayed in Figure 2. Model #4 of the PROCESS macro is composed of submodels. Submodels 1 and 2 involve regressing each mediator—namely diet quality or protein intake and physical activity—onto sleep using simple linear regression. Paths A and D in Figure 2 show where diet quality or protein intake and physical activity are regressed onto sleep (quality or duration), respectively. Submodel 3 involves regressing HG/BMI onto sleep (Path C). The last submodel of this PROCESS macro includes 5 direct effects, displayed in Figure 2 as Paths A–E. Two specific indirect effects are computed as the product of path coefficients (Path A × Path B; Path D × Path E). The PROCESS macro also allowed for the exploration of the interaction between sleep quality or duration and the mediators. The study predictors, outcome, and diet quality mediators were continuous variables while Life Simple 7 was categorical. As stated earlier, all models were adjusted for the covariates. The unstandardized b (or beta) coefficients were generated from the PROCESS macro. In PROCESS, the indirect effect is bootstrapped to 95% confidence intervals. If zero does not fall within these confidence intervals, the indirect effect is significant at *p* < 0.05.

## 3. Results

### 3.1. The Characteristics of the Sample

#### 3.1.1. Overall Sample Characteristics

The mean age of the sample was 56 years (Table 1). Approximately 58% of the sample were women and 42% were White individuals. While roughly 41% were current smokers, 3 out of 4 individuals had either hypertension, diabetes, and/or metabolic syndrome. The number of persons with metabolic syndrome equaled 308 (24.5%); diabetes, 271 (20.7%); pre-diabetes, 214 (16.4%); and hypertension, 802 (61.3%).

The mean PSQI score was 7.4 with 61% of the sample’s sleep quality rated as poor based on a score of >5 (Table 1). In contrast, 34% of the sample rated their sleep quality as fairly or very bad. The mean hours of sleep per night was 6 h. Only 18% of the sample achieved the Life Simple 7 goal of 150 min of activity per week. The scores for each of the 7 components of the PSQI are provided in Table 1. The summary of the reasons for troubled sleep during the past month (the sleep disturbance component) is provided in Supplementary Table S1. Over half of the sample reported waking up in the middle of the night or early in the morning or having to get up to use the bathroom 3 or more times per week.

The mean (±SE) handgrip strength of the sample was 32.3 ± 0.3 kg. Men had a significantly greater mean handgrip strength than women (40.9 ± 0.4 kg and 26.0 ± 0.3 kg, respectively, *p* < 0.001). The mean HG/BMI was 1.14 ± 0.13 (Table 1).

As shown in Table 1, the mean daily energy intake of the sample was 1978 kcal, with men and women averaging 2312 ± 38 kcal and 1739 ± 24 kcal, respectively. The mean protein intake per body weight of men was 1.04 ± 0.02 g/kg and of women was 0.81 ± 0.01 g/kg. The mean HEI-2010 score of the sample was 49 out of 100 with a range of 14 to 90. The mean e-DII was 5.17 ± 0.03, ranging from −0.63 to 7.27.

#### 3.1.2. Characteristics of Sample Categorized by Sleep Quality

The mean PSQI score for those with good sleep quality was 3.2 ± 0.06, a score significantly lower than those with poor quality sleep (10.11 ± 0.12) (Table 1). The mean hours of sleep per night was significantly greater for those with good quality sleep compared to those with poor quality sleep, 7.0 vs. 5.2 h, respectively. As presented in Table 1, the scores for all of the 7 components of the PSQI significantly differed by sleep quality group. Handgrip strength/BMI, HEI-2010 scores, and mean protein intake per kg of body weight of the poor-quality sleepers were lower than those of the good-quality sleepers (Table 2). The inflammatory potential of their diets was also higher. Approximately 14% of the sample with poor sleep quality compared to about 26% of the sample with good sleep quality achieved the Life Simple 7 goal of 150 min of activity per week.

### 3.2. Summary of Mediational Analyses

#### 3.2.1. Sleep Quality

The findings of the PROCESS macro, model #4 using sleep quality are shown in Table 2, Table 3 and Table 4. Sleep quality predicted all 3 diet measures (Path A), physical activity (Path D), and HG/BMI (Path C). Of the remaining two direct effects (Paths B and E), only two measures of diet quality were found to be significant. HEI-2010 and protein intake/kg of body weight predicted HG/BMI (Path B). The only mediator of the relationship between sleep quality and HG/BMI was protein intake per body weight, g/kg (Table 4). There were no significant interactions between sleep quality and any mediator.

#### 3.2.2. Sleep Duration

The findings of the PROCESS macro, model #4 using sleep duration are provided in Table 5, Table 6 and Table 7. Of the five direct effects, the relationship between sleep duration and HG/BMI, Path C, was always nonsignificant. Sleep duration predicted HEI-2010 and protein intake per kg of body weight (Path A) (Table 6 and Table 7). Sleep duration only predicted physical activity (Path D) when e-DII was used as the diet quality measure (Table 5). Only HEI-2010 and protein intake per body weight, g/kg, directly and significantly predicted HG/BMI (Path B) (Table 5, Table 6 and Table 7). Physical activity predicted HG/BMI (Path E) only when e-DII was used as the diet quality measure (Table 5). Protein intake per body weight (g/kg) and HEI-2010 were mediators of the relationship between sleep duration and HG/BMI. (Table 6 and Table 7). The interaction of e-DII with hours slept was marginally significant (*p* = 0.05).

#### 3.2.3. Significant Covariates

The complete results of the PROCESS macro, model #4 for both sleep quality and sleep duration are provided in Supplementary Tables S2–S7. Four covariates were significant in all the models for both sleep quality and sleep duration. Being of a younger age and male were significantly associated with greater handgrip strength at *p* < 0.0001. Individuals with medical conditions and nonsmokers had lower handgrip strength compared to those without medical conditions (*p* < 0.0001) and those who smoked cigarettes (*p* ranged from 0.002 to <0.0001), respectively.

## 4. Discussion

The study findings, to our knowledge, are the first to document the role of protein as a mediator in the relationship between sleep quality and sleep duration with handgrip strength in middle-aged White and African American adults. Additionally, this study found that the Healthy Eating Index-2010 score, a proxy for overall diet quality, was a mediator of the relationship between sleep duration and handgrip strength. These findings might be explained because adequate protein intake, which provides the essential amino acids for skeletal muscle growth and maintenance, is part of a healthy dietary pattern. These results emphasize the importance of consuming a diet with more healthful foods and adequate quantities of protein for preserving muscle strength with age. Furthermore, both HEI-2010 and protein intake positively predicted handgrip strength. The influence of protein intake on handgrip strength has been reported [19,21,22,23].

Our study contributes to the literature with the finding that sleep quality and also sleep duration positively and directly impact protein intake. The findings of the positive and direct effects of sleep quality and longer sleep duration on diet quality, assessed by the HEI, were consistent with the publications of other researchers [50,51]. Poor sleep quality and short sleep duration can influence food choices. Sleep restriction can trigger the endocannabinoid system, increasing the desire to eat as well as the pleasure and satisfaction gained from eating, and can decrease leptin levels [52,53]. Evidence exists that people whose sleep is restricted tend to consume unhealthy foods, especially snacks and desserts [13,17,50] and more dietary energy [54,55]. The differences in the results by diet quality methods can be attributed to their components. In fact, the HEI and DII have different focus areas, with the HEI focusing on overall diet quality and nutritional adequacy, while the DII targets inflammatory potential [36]. The HEI-2010 may show high scores for an adequate diet, while the DII may highlight subtle differences in nutrient intake driving inflammation [56]. Both have implications for health outcomes, with the HEI-2010 relating diet quality to cardiovascular health and the DII providing stronger associations with inflammatory-related conditions [57]. Zare and colleagues also reported no association between the e-DII and sleep quality [58].

Physical activity was not a mediator of any relationship between sleep and handgrip strength. Handgrip strength has been associated with mild cognitive impairment in a mediation model with moderate to vigorous physical activity [59]. There are many complexities in physical activity measures including the frequency, intensity, type, and duration of the activity. The linkages between physical activity and handgrip strength are dependent on the mode of activity [32]. In this study, physical activity was measured by the Life Simple 7, an approach that may not have captured specific modes like occupation-like activity, as well as the intensity of physical activity, that may impact handgrip strength. The direct association of sleep quality and sleep duration with physical activity has been supported by other researchers [26,27,60]. Physical activity has been associated with higher sleep quality in community-dwelling older US adults [27]. In young adults, physical activity influences sleep duration positively [26]. In older United Kingdom women, poor sleep quality identified by the PSQI has been associated with poor handgrip strength [60].

Healthy lifestyle behaviors are universally recognized as key to promoting health and reducing disease risk with age. These behaviors include consuming a healthy diet, limiting alcohol, engaging in regular moderate-to-vigorous physical activity, getting enough and high-quality sleep, achieving and maintaining a healthy weight (BMI < 25), avoiding tobacco, and managing stress. Based on the characteristics of the overall study sample, many participants, especially those with poor-quality sleep, need to make improvements in their behaviors. Approximately 41% smoked cigarettes at the time of the study compared to 17.8% of all US persons ≥ 18 years of age in 2013 [61]. The mean number of hours slept was lower than the recommended duration, with 61% of the sample receiving a poor sleep quality rating on the PSQI. Based on the e-DII and the HEI-2010 scores, their dietary patterns were proinflammatory and only aligned with less than half of the *DGA*, respectively. The mean HEI-2010 score of the HANDLS study sample was approximately 10 points lower than that of a nationally represented US population (WWEIA-NHANES 2010–2011, HEI-2010 = 58.27) [62]. The mean protein intake of the sample exceeded 0.8 g/kg body weight/day, the Recommended Dietary Allowance (RDA) for protein [63]. However, this value is considered by some to be too low to have beneficial effects on muscle mass [22,64]. In our sample, roughly one in five persons engaged in the American Heart Association’s recommended physical activity level. Yet, the mean dominant handgrip strength of the sample was comparable to that of US men and women, 55–59 years of age [65]. Improvements in food choices and engagement in more activity could increase the handgrip strength of the sample participants, which would be beneficial to their overall health as they age.

Handgrip strength is considered to be an indicator of physical capability [4,66]. It is associated with multiple health outcomes and can be modified by several factors [67]. We found that handgrip strength was significantly correlated with physical performance measures such as chair stands, single-leg stands, and the tandem stand. Among the covariates, men and younger individuals had significantly greater handgrip strength than women and older aged persons, respectively. These findings are consistent with those of other researchers [68,69,70]. Almashaqbeh and colleagues reported that men had significantly greater handgrip strength than women regardless of the anatomical position of their arm when the measurement was taken [68]. A systematic review of 117 longitudinal studies revealed that increasing age was associated with a decline in handgrip strength [70]. The handgrip strength of nonsmokers and persons with medical conditions was significantly lower than that of “healthier” persons and smokers, respectively. The inverse relationship between smoking and handgrip strength is most likely attributed to the lower BMI of smokers. This relationship between smokers and handgrip strength is consistent with the findings of Cho and colleagues, based on data from the Korean National Nutrition and Health Examination Survey [71].

One of the many strengths of the study is the measures used in these analyses. The dietary measures were based on 2 days of 24 h recalls collected from a validated method which may be unique to longitudinal population-based studies. Both dietary quality measures are considered valid and reliable. The collection of sleep and physical activity data used validated methods. Other strengths include the combination of self-reported diet and activity measures with actual measures of muscle strength and the adjustments for many factors affecting handgrip strength in the analyses. The PROCESS macro is a powerful tool for statistical mediation analysis that allows one to customize models to test complex models with multiple mediators and also their interactions with the independent variable [48,49]. The community-dwelling sample consists of men and women with different socioeconomic and racial profiles who are underrepresented in the nutrition literature.

There are also limitations. The sample is relatively small and not representative of the national population like NHANES. As with any self-reported data, systematic biases can exist. Self-reported sleep duration has been shown to overestimate objectively measured sleep across all races [72]. Overestimation was significantly higher in Whites compared to Blacks [72]. Objective measures of sleep quality like polysomnography and actigraphy were not available, but they are moderately correlated to PSQI scores [73]. Estimations of food portions consumed can be over- or underestimated. Individuals with obesity have been found to underreport energy while individuals who are overweight overreport energy [74]. Dietary recalls can also be affected by persons’ concerns about reporting socially undesirable foods. Self-reported physical activity can also differ from activity measured by accelerometers. Researchers have found that persons self-report more vigorous activity and less sedentary time compared with the accelerometer [75]. The difference between these methods can be affected by sex, age, and education [75]. These reporting biases could reduce the ability to identify significant associations between sleep and handgrip strength. The analysis is cross-sectional, so no causality can be established. Despite these limitations, the significant findings on mediators can be a starting point for future research with larger sample sizes to confirm the results.

## 5. Conclusions

This study’s findings revealed the mediating roles of diet quality, assessed by the Healthy Eating Index, and protein intake expressed as g per kg of body weight on the relationship between sleep and handgrip strength. This study further expands our knowledge of the relationship between sleep quality and sleep duration and handgrip strength among middle-aged adults, emphasizing the importance of the consumption of protein-rich foods as part of a healthy diet.

## Figures and Tables

**Table 1:** The characteristics of the overall HANDLS sample and by category of sleep quality ^a^.

Characteristic	Overall	Sleep Quality	
	*n* = 1308	Good *n* = 512	Poor *n* = 796
Age, y, X ± SE	56.3 ± 0.3	56.3 ± 0.4	56.4 ± 0.3
Sex, % men	41.6	39.4	45.3
Race, % African American	58.1	60.0	57.7
Poverty, % <125%	36.8	34.2	39.6
Cigarette smoker, %	40.9	35.4	47.8
Drug user, %	85.2	85.8	83.5
Medical condition, %	73.5	66.6	75.4
*Sleep*			
PSQI global score, X ± SE	7.39 ± 0.12	3.2 ± 0.06	10.11 ± 0.12 ***
Quality, % poor (PSQI > 5)	60.9	-	-
PSQI Sleep disturbance component, X ± SE	1.29 ± 0.02	0.96 ± 0.02	1.49 ± 0.02 ***
PSQI Sleep daytime dysfunction component, X ± SE	0.67 ± 0.03	0.28 ± 0.03	0.92 ± 0.04 ***
PSQI Sleep duration component, X ± SE	1.28 ± 0.03	0.49 ± 0.03	1.79 ± 0.04 ***
PSQI Sleep efficiency component, X ± SE	1.07 ± 0.03	0.28 ± 0.03	1.59 ± 0.04 ***
PSQI Sleep latency component, X ± SE	1.30 ± 0.04	0.48 ± 0.04	1.83 ± 0.04 ***
PSQI Sleep medication component, X ± SE	0.55 ± 0.03	0.12 ± 0.02	0.83 ± 0.04 ***
PSQI Sleep subjective quality component, X ± SE	1.21 ± 0.03	0.54 ± 0.03	1.65 ± 0.03 ***
Self-reported duration, hours/night, X ± SE	5.9 ± 0.05	7.0 ± 0.05	5.2 ± 0.05 ***
*Self-reported sleep quality:*			
Very good, %	25.6	52.0	8.7
Fairly good, %	39.9	42.8	38.1
Fairly bad, %	21.9	4.9	32.9
Very bad, %	12.5	0.4	20.4
Handgrip/BMI, kg, X ± SE	1.14 ± 0.13	1.20 ± 0.02	1.10 ± 0.02 ***
*Diet*			
e-DII score, X ± SE	5.17 ± 0.03	5.03 ± 0.06	5.26 ± 0.04 **
HEI-2010 score, X ± SE	48.53 ± 0.34	50.3 ± 0.6	47.4 ± 0.4 ***
Protein intake/body weight, g/kg, X ± SE	0.91 ± 0.01	0.96 ± 0.02	0.88 ± 0.02 **
Energy, kcal, X ± SE	1978 ± 23	2045 ± 38	1936 ± 28 *
*Physical Activity*			*p* < 0.001
No physical activity, %	13.9	9.8	16.6
<150 min activity/week, %	67.7	64.5	69.7
≥150 min activity/week, %	18.4	25.8	13.7

^a^ Quality determined by Pittsburgh Sleep Quality Index. * *p* < 0.05; ** *p* < 0.01; *** *p* < 0.001. Abbreviations: BMI—body mass index; e-DII—energy-adjusted Dietary Inflammatory Index; HEI—Healthy Eating Index; PSQI—Pittsburgh Sleep Quality Index; X ± SE—mean ± standard error.

**Table 2:** The mediation analysis summary of the effects of the energy-adjusted Dietary Inflammatory Index (e-DII) and Life Simple 7 (LS7) physical activity on the relationship between sleep quality and handgrip strength based on the Hayes Process macro, model #4 ^a^.

Path: Relationships	Direct Effect ^b^	*p*		
A: Sleep quality → e-DII	0.029 ± 0.009	0.0006		
D: Sleep quality → LS7	0.022 ± 0.004	<0.0001		
C: Sleep quality → HG/BMI	−0.007 ± 0.002	0.006		
B: e-DII → HG/BMI	−0.002 ± 0.009	0.062		
E: LS7 → HG/BMI	−0.037 ± 0.019	0.071		
**Outcome: HG/BMI**	**Indirect Effect**		**Bootstrapped Confidence Interval**	
			Lower Bound	Upper Bound
Sleep quality → e-DII → HG/BMI	−0.0001 ± 0.0003	NS	−0.0006	0.0005
Sleep quality → LS7 → HG/BMI	−0.0008 ± 0.0005	NS	−0.0018	0.0001

^a^ The PROCESS model adjusted for age, race, sex, poverty status, smoking, drug use, and medical conditions. Total effect model R^2^ = 0.49. ^b^ Beta coefficients ± standard error. Abbreviations: e-DII—energy-adjusted Dietary Inflammatory Index; HG/BMI—handgrip/body mass index; LS7—Life Simple 7; NS—nonsignificant.

**Table 3:** The mediation analysis summary of the effects of the Healthy Eating Index (HEI)-2010 and Life Simple 7 (LS7) physical activity on the relationship between sleep quality and handgrip strength based on the Hayes Process macro, model #4 ^a^.

Path: Relationships	Direct Effect ^b^	*p*		
A: Sleep quality → HEI	−0.318 ± 0.080	0.0001		
D: Sleep quality → LS7	0.022 ± 0.004	<0.0001		
C: Sleep quality → HG/BMI	−0.006 ± 0.002	0.009		
B: HEI-2010 → HG/BMI	0.002 ± 0.001	0.038		
E: LS7 → HG/BMI	−0.031 ± 0.019	0.107		
**Outcome: HG/BMI**	**Indirect Effect**		**Bootstrapped Confidence Interval**	
			Lower Bound	Upper Bound
Sleep quality → HEI → HG/BMI	−0.0006 ± 0.0003	NS	−0.0013	0.0000
Sleep quality → LS7 → HG/BMI	−0.0007 ± 0.0005	NS	−0.0017	0.0002

^a^ The PROCESS model adjusted for age, race, sex, poverty status, smoking, drug use, and medical conditions. Total effect model R^2^ = 0.49. ^b^ Beta coefficients ± standard error. Abbreviations: HEI—Healthy Eating Index; HG/BMI—handgrip/body mass index; LS7—Life Simple 7; NS—nonsignificant.

**Table 4:** The mediation analysis summary of the effects of protein intake/body weight (Pr/BW, g/kg) and Life Simple 7 (LS7) physical activity on the relationship between sleep quality and handgrip strength based on the Hayes Process macro, model #4 ^a^.

Path: Relationships	Direct Effect ^b^	*p*		
A: Sleep quality→ Pr/BW	−0.006 ± 0.002	0.007		
D: Sleep quality → LS7	0.022 ± 0.004	<0.001		
C: Sleep quality → HG/BMI	−0.005 ± 0.002	0.034		
B: Pr/BW → HG/BMI	0.275 ± 0.030	<0.0001		
E: LS7 → HG/BMI	−0.027 ± 0.018	0.144		
**Outcome: HG/BMI**	**Indirect Effect**		**Bootstrapped Confidence Interval**	
			Lower Bound	Upper Bound
Sleep quality → Pr/BW → HG/BMI	−0.0017 ± 0.0006	<0.05	−0.0030	−0.0006
Sleep quality → LS7 → HG/BMI	−0.006 ± 0.0004	NS	−0.0015	0.0002

^a^ The PROCESS model adjusted for age, race, sex, poverty status, mean energy, smoking, drug use, and medical conditions. Total effect model R^2^ = 0.49. ^b^ Beta coefficients ± standard error. Abbreviations: HG/BMI—handgrip/body mass index; LS7—Life Simple 7; NS—nonsignificant; Pr/BW—protein/body weight, g/kg.

**Table 5:** The mediation analysis summary of the effects of the energy-adjusted Dietary Inflammatory Index (e-DII) and Life Simple 7 (LS7) on the relationship between sleep duration and handgrip strength based on the Hayes Process macro, model #4 ^a^.

Path: Relationships	Direct Effect ^b^	*p*		
A: Sleep duration → e-DII	−0.027 ± 0.023	0.237		
D: Sleep duration → LS7	−0.039 ± 0.010	0.0001		
C: Sleep duration → HG/BMI	0.009 ± 0.006	0.178		
B: e-DII → HG/BMI	−0.004 ± 0.009	0.664		
E: LS7 → HG/BMI	−0.043 ± 0.019	0.025		
**Outcome: HG/BMI**	**Indirect Effect**		**Bootstrapped Confidence Interval**	
			Lower Bound	Upper Bound
Sleep duration → e-DII → HG/BMI	0.0001 ± 0.0003	NS	−0.0004	0.0008
Sleep duration → LS7 → HG/BMI	0.0017 ± 0.0009	NS	0.0001	0.0037

^a^ The PROCESS model adjusted for age, race, sex, poverty status, smoking, drug use, and medical conditions. Total effect model R^2^ = 0.49. ^b^ Beta coefficients ± standard error. Abbreviations: e-DII—energy-adjusted Dietary Inflammatory Index; HG/BMI—handgrip/body mass index; LS7—Life Simple 7; NS—nonsignificant.

**Table 6:** The mediation analysis summary of the effects of the Healthy Eating Index (HEI)-2010 and Life Simple 7 (LS7) on the relationship between sleep duration and handgrip strength based on the Hayes Process macro, model #4 ^a^.

Path: Relationships	Direct Effect ^b^	*p*		
A: Sleep duration → HEI	0.627 ± 0.212	0.0032		
D: Sleep duration → LS7	−0.040 ± 0.010	0.0001		
C: Sleep duration → HG/BMI	0.008 ± 0.006	0.228		
B: HEI-2010 → HG/BMI	0.002 ± 0.001	0.026		
E: LS7 → HG/BMI	−0.036 ± 0.019	0.060		
**Outcome: HG/BMI**	**Indirect Effect**		**Bootstrapped Confidence Interval**	
			Lower Bound	Upper Bound
Sleep duration → HEI → HG/BMI	0.0013 ± 0.0007	<0.05	0.0001	0.0030
Sleep duration → LS7 → HG/BMI	0.0014 ± 0.0009	NS	−0.0001	0.0034

^a^ The PROCESS model adjusted for age, race, sex, poverty status, smoking, drug use, and medical conditions. Total effect model R^2^ = 0.49. ^b^ Beta coefficients ± standard error. Abbreviations: HEI—Healthy Eating Index; HG/BMI—handgrip/body mass index; LS7—Life Simple 7; NS—nonsignificant.

**Table 7:** The mediation analysis summary of the effects of protein intake/body weight (Pr/BW, g/kg) and Life Simple 7 (LS7) on the relationship between sleep duration and handgrip strength based on the Hayes Process macro, model #4 ^a^.

Path: Relationships	Direct Effect ^b^	*p*		
A: Sleep duration → Pr/BW	0.021 ± 0.006	0.0008		
D: Sleep duration → LS7	−0.040 ± 0.010	0.0001		
C: Sleep duration → HG/BMI	0.010 ± 0.006	0.110		
B: Pr/BW → HG/BMI	0.278 ± 0.030	<0.0001		
E: LS7 → HG/BMI	−0.032 ± 0.018	0.079		
**Outcome: HG/BMI**	**Indirect Effect**		**Bootstrapped Confidence Interval**	
			Lower Bound	Upper Bound
Sleep duration → Pr/BW → HG/BMI	0.0057 ± 0.0019	<0.05	0.0023	0.0098
Sleep duration → LS7 → HG/BMI	0.0013 ± 0.0009	NS	−0.0003	0.0032

^a^ The PROCESS model adjusted for age, race, sex, poverty status, mean energy, smoking, drug use, and medical conditions. Total effect model R^2^ = 0.49. ^b^ Beta coefficients ± standard error. Abbreviations: HG/BMI—handgrip/body mass index; LS7—Life Simple 7; NS—nonsignificant; Pr/BW—protein/body weight, g/kg.

## Data Availability

Data are available upon request to researchers with valid proposals who agree to the confidentiality agreement as required by our Institutional Review Board. We publicize our policies on our website https://handls.nih.gov (accessed on 16 January 2025). Requests for data access may be sent to Alan Zonderman (co-author) or the study manager, Jennifer Norbeck, at norbeckje@mail.nih.gov.

## Supplementary Materials

The following supporting information can be downloaded at: https://www.mdpi.com/article/10.3390/nu17111900/s1, Table S1. Summary of Pittsburgh Sleep Quality Index responses to component 5; Table S2. Output for Process macro, Model #4 with sleep quality and handgrip strength incorporating energy-adjusted Dietary Inflammatory Index (e-DII), physical activity, and covariates; Table S3. Output for Process macro, Model #4 with sleep quality and handgrip strength incorporating Healthy Eating Index-2010 (HEI) and physical activity; Table S4. Output for Process macro, Model #4 with sleep quality and handgrip strength incorporating protein intake (g)/kg of body weight (protein/BW) and physical activity; Table S5. Output for Process macro, Model #4 with sleep duration and handgrip strength incorporating energy-adjusted Dietary Inflammatory Index (e-DII) and physical activity; Table S6. Output for Process macro, Model #4 with sleep duration and handgrip strength incorporating Healthy Eating Index-2010 (HEI) and physical activity; Table S7. Output for Process macro, Model #4 with sleep duration and handgrip strength incorporating protein intake (g)/kg of body weight (protein/BW) and physical activity.

## Abbreviations

The following abbreviations are used in this manuscript:

DGA	Dietary Guidelines for Americans
e-DII	Energy-Adjusted Dietary Inflammatory Index
HANDLS	Healthy Aging in Neighborhoods of Diversity across the Life Span
HEI	Healthy Eating Index
HG/BMI	Handgrip Strength/Body Mass Index
LS7	Life Simple 7
NS	Nonsignificant
Pr/BW	Protein Intake/Body Weight (g/kg)
PSQI	Pittsburgh Sleep Quality Index
SE	Standard Error
USDA	United States Department of Agriculture

## References

[1] Ramar K., Malhotra R.K., Carden K.A., Martin J.L., Abbasi-Feinberg F., Aurora R.N., Kapur V.K., Olson E.J., Rosen C.L., Rowley J.A. (2021). Sleep is essential to health: An American Academy of Sleep Medicine position statement. J. Clin. Sleep Med..

[2] National Center for Health Statistics (2022). QuickStats: Percentage of Adults Aged ≥ 18 Years Who Sleep <7 Hours on Average in a 24-Hour Period, by Sex and Age Group—National Health Interview Survey, United States, 2020. MMWR Morb. Mortal. Wkly. Rep..

[3] St-Onge M.P., Cherta-Murillo A., Darimont C., Mantantzis K., Martin F.P., Owen L. (2023). The interrelationship between sleep, diet, and glucose metabolism. Sleep Med. Rev..

[4] Vaishya R., Misra A., Vaish A., Ursino N., D’Ambrosi R. (2024). Hand grip strength as a proposed new vital sign of health: A narrative review of evidences. J. Health Popul. Nutr..

[5] Hamasaki H. (2019). The association between handgrip strength and sleep duration in Japanese patients with type 2 diabetes. Diabetes Metab..

[6] Liu J., Zhang T., Luo J., Chen S., Zhang D. (2023). Association between Sleep Duration and Grip Strength in U.S. Older Adults: An NHANES Analysis (2011–2014). Int. J. Environ. Res. Public Health.

[7] Pana A., Sourtzi P., Kalokairinou A., Pastroudis A., Chatzopoulos S.T., Velonaki V.S. (2021). Association between muscle strength and sleep quality and duration among middle-aged and older adults: A systematic review. Eur. Geriatr. Med..

[8] Lee G., Baek S., Park H.W., Kang E.K. (2018). Sleep Quality and Attention May Correlate with Hand Grip Strength: FARM Study. Ann. Rehabil. Med..

[9] Laredo-Aguilera J.A., Carmona-Torres J.M., Cobo-Cuenca A.I., Garcia-Pinillos F., Latorre-Roman P.A. (2019). Handgrip Strength is Associated with Psychological Functioning, Mood and Sleep in Women over 65 Years. Int. J. Environ. Res. Public Health.

[10] Zuraikat F.M., St-Onge M.-P., Watson R.R., Preedy V.R. (2020). Chapter 22—The Influence of Diet on Sleep. Neurological Modulation of Sleep.

[11] Pot G.K. (2018). Sleep and dietary habits in the urban environment: The role of chrono-nutrition. Proc. Nutr. Soc..

[12] Godos J., Grosso G., Castellano S., Galvano F., Caraci F., Ferri R. (2021). Association between diet and sleep quality: A systematic review. Sleep Med. Rev..

[13] Dashti H.S., Scheer F.A., Jacques P.F., Lamon-Fava S., Ordovas J.M. (2015). Short sleep duration and dietary intake: Epidemiologic evidence, mechanisms, and health implications. Adv. Nutr..

[14] Zhou J., Kim J.E., Armstrong C.L., Chen N., Campbell W.W. (2016). Higher-protein diets improve indexes of sleep in energy-restricted overweight and obese adults: Results from 2 randomized controlled trials. Am. J. Clin. Nutr..

[15] Wilson K., St-Onge M.P., Tasali E. (2022). Diet Composition and Objectively Assessed Sleep Quality: A Narrative Review. J. Acad. Nutr. Diet..

[16] Burrows T., Fenton S., Duncan M. (2020). Diet and sleep health: A scoping review of intervention studies in adults. J. Hum. Nutr. Diet..

[17] St-Onge M.P., Grandner M.A., Brown D., Conroy M.B., Jean-Louis G., Coons M., Bhatt D.L. (2016). Sleep Duration and Quality: Impact on Lifestyle Behaviors and Cardiometabolic Health: A Scientific Statement from the American Heart Association. Circulation.

[18] Shahdadian F., Boozari B., Saneei P. (2023). Association between short sleep duration and intake of sugar and sugar-sweetened beverages: A systematic review and meta-analysis of observational studies. Sleep Health.

[19] Fanelli Kuczmarski M., Pohlig R.T., Stave Shupe E., Zonderman A.B., Evans M.K. (2018). Dietary Protein Intake and Overall Diet Quality Are Associated with Handgrip Strength in African American and White Adults. J. Nutr. Health Aging.

[20] Kuczmarski M.F., Beydoun M.A., Zonderman A.B., Evans M.K. (2022). Intakes of Total and Branched-Chain Essential Amino Acids are Positively Associated with Handgrip Strength in African American and White Urban Younger and Older Adults. J. Nutr. Gerontol. Geriatr..

[21] Pikosky M.A., Cifelli C.J., Agarwal S., Fulgoni III V.L. (2022). Association of Dietary Protein Intake and Grip Strength Among Adults Aged 19+ Years: NHANES 2011–2014 Analysis. Front. Nutr..

[22] Kim M.H., Choi M.K., Bae Y.J. (2023). Relationship between protein intake and grip strength in qualitative and quantitative aspects among the elderly in Korea: Results from the Korea National Health and Nutrition Examination Survey. BMC Geriatr..

[23] Jun S., Cowan A.E., Dwyer J.T., Campbell W.W., Thalacker-Mercer A.E., Gahche J.J., Bailey R.L. (2021). Dietary Protein Intake Is Positively Associated with Appendicular Lean Mass and Handgrip Strength among Middle-Aged US Adults. J. Nutr..

[24] Alnawwar M.A., Alraddadi M.I., Algethmi R.A., Salem G.A., Salem M.A., Alharbi A.A. (2023). The Effect of Physical Activity on Sleep Quality and Sleep Disorder: A Systematic Review. Cureus.

[25] Atoui S., Chevance G., Romain A.J., Kingsbury C., Lachance J.P., Bernard P. (2021). Daily associations between sleep and physical activity: A systematic review and meta-analysis. Sleep Med. Rev..

[26] Mead M.P., Baron K., Sorby M., Irish L.A. (2019). Daily Associations Between Sleep and Physical Activity. Int. J. Behav. Med..

[27] Dzierzewski J.M., Buman M.P., Giacobbi P.R., Roberts B.L., Aiken-Morgan A.T., Marsiske M., McCrae C.S. (2014). Exercise and sleep in community-dwelling older adults: Evidence for a reciprocal relationship. J. Sleep Res..

[28] de Lima T.R., Silva D.A.S., de Castro J.A.C., Christofaro D.G.D. (2017). Handgrip strength and associated sociodemographic and lifestyle factors: A systematic review of the adult population. J. Bodyw. Mov. Ther..

[29] McGrath R.P., Kraemer W.J., Snih S.A., Peterson M.D. (2018). Handgrip Strength and Health in Aging Adults. Sports Med..

[30] McLeod M., Breen L., Hamilton D.L., Philp A. (2016). Live strong and prosper: The importance of skeletal muscle strength for healthy ageing. Biogerontology.

[31] Rantanen T., Guralnik J.M., Foley D., Masaki K., Leveille S., Curb J.D., White L. (1999). Midlife hand grip strength as a predictor of old age disability. JAMA.

[32] Labott B.K., Bucht H., Morat M., Morat T., Donath L. (2019). Effects of Exercise Training on Handgrip Strength in Older Adults: A Meta-Analytical Review. Gerontology.

[33] Bilajac L., Juraga D., Žuljević H., Glavić M.M., Vasiljev V., Rukavina T. (2019). The influence of physical activity on handgrip strength of elderly. Arch. Gerontol. Geriatr. Res..

[34] Evans M.K., Lepkowski J.M., Powe N.R., LaVeist T., Kuczmarski M.F., Zonderman A.B. (2010). Healthy aging in neighborhoods of diversity across the life span (HANDLS): Overcoming barriers to implementing a longitudinal, epidemiologic, urban study of health, race, and socioeconomic status. Ethn. Dis..

[35] Buysse D.J., Reynolds C.F., Monk T.H., Berman S.R., Kupfer D.J. (1989). The Pittsburgh sleep quality index: A new instrument for psychiatric practice and research. Psychiatry Res..

[36] DiNatale J.C., Azarmanesh D., Hébert J.R., Wirth M.D., Pearlman J., Crowe-White K.M. (2023). Relationship between Non-Energy-Adjusted and Energy-Adjusted Dietary Inflammatory Index and the Healthy Eating Index-2015: An analysis of the National Health and Nutrition Examination Survey (NHANES) 2015–2018. Ann. Med..

[37] Shivappa N., Steck S.E., Hurley T.G., Hussey J.R., Hebert J.R. (2014). Designing and developing a literature-derived, population-based dietary inflammatory index. Public Health Nutr..

[38] Zheng D., Zhuo B., Zheng G., Hua J., Zhang J., Wang C., Wang Y., Zhang Z., Lin H. (2023). The associations of energy adjusted dietary inflammatory index with brain structure and cognitive function. Innov. Med..

[39] Guenther P.M., Kirkpatrick S.I., Reedy J., Krebs-Smith S.M., Buckman D.W., Dodd K.W., Casavale K.O., Carroll R.J. (2014). The Healthy Eating Index-2010 is a valid and reliable measure of diet quality according to the 2010 Dietary Guidelines for Americans. J. Nutr..

[40] Moshfegh A.J., Rhodes D.G., Baer D.J., Murayi T., Clemens J.C., Rumpler W.V., Paul D.R., Sebastian R.S., Kuczynski K.J., Ingwersen L.A. (2008). The US Department of Agriculture Automated Multiple-Pass Method reduces bias in the collection of energy intakes. Am. J. Clin. Nutr..

[41] US Department of Agriculture Food and Nutrient Database for Dietary Studies. https://www.ars.usda.gov/northeast-area/beltsville-md-bhnrc/beltsville-human-nutrition-research-center/food-surveys-research-group/docs/fndds-download-databases/.

[42] Baecke J.A., Burema J., Frijters J.E. (1982). A short questionnaire for the measurement of habitual physical activity in epidemiological studies. Am. J. Clin. Nutr..

[43] Beydoun M.A., Georgescu M.F., Hossain S., Beydoun H.A., Fanelli-Kuczmarski M.T., Evans M.K., Zonderman A.B. (2023). Life’s simple 7 and its association with trajectories in depressive symptoms among urban middle-aged adults. J. Affect. Disord..

[44] Hasbani N.R., Ligthart S., Brown M.R., Heath A.S., Bebo A., Ashley K.E., Boerwinkle E., Morrison A.C., Folsom A.R., Aguilar D. (2022). American Heart Association’s Life’s Simple 7: Lifestyle Recommendations, Polygenic Risk, and Lifetime Risk of Coronary Heart Disease. Circulation.

[45] US Department of Health and Human Services The 2004 HHS Poverty Guidelines. https://aspe.hhs.gov/2004-hhs-poverty-guidelines.

[46] Mainous A.G., Tanner R.J., Anton S.D., Jo A. (2015). Grip Strength as a Marker of Hypertension and Diabetes in Healthy Weight Adults. Am. J. Prev. Med..

[47] Wen Y., Liu T., Ma C., Fang J., Zhao Z., Luo M., Xia Y., Zhao Y., Ji C. (2022). Association between handgrip strength and metabolic syndrome: A meta-analysis and systematic review. Front. Nutr..

[48] Hayes A.F. (2022). Introduction to Mediation, Moderation, and Conditional Process Analysis: A Regression-Based Approach.

[49] Hayes A.F., Montoya A.K., Rockwood N.J. (2017). The Analysis of Mechanisms and Their Contingencies: PROCESS versus Structural Equation Modeling. Australas. Mark. J..

[50] Theorell-Haglöw J., Lemming E.W., Michaëlsson K., Elmståhl S., Lind L., Lindberg E. (2020). Sleep duration is associated with healthy diet scores and meal patterns: Results from the population-based EpiHealth study. J. Clin. Sleep Med..

[51] Stefan L., Radman I., Podnar H., Vrgoc G. (2018). Sleep Duration and Sleep Quality Associated with Dietary Index in Free-Living Very Old Adults. Nutrients.

[52] Spiegel K., Tasali E., Penev P., Van Cauter E. (2004). Brief communication: Sleep curtailment in healthy young men is associated with decreased leptin levels, elevated ghrelin levels, and increased hunger and appetite. Ann. Intern. Med..

[53] Cedernaes J., Fanelli F., Fazzini A., Pagotto U., Broman J.-E., Vogel H., Dickson S.L., Schiöth H.B., Benedict C. (2016). Sleep restriction alters plasma endocannabinoids concentrations before but not after exercise in humans. Psychoneuroendocrinology.

[54] Stern J.H., Grant A.S., Thomson C.A., Tinker L., Hale L., Brennan K.M., Woods N.F., Chen Z. (2014). Short sleep duration is associated with decreased serum leptin, increased energy intake and decreased diet quality in postmenopausal women. Obesity.

[55] Spaeth A.M., Dinges D.F., Goel N. (2014). Sex and race differences in caloric intake during sleep restriction in healthy adults. Am. J. Clin. Nutr..

[56] Wirth M.D., Hebert J.R., Shivappa N., Hand G.A., Hurley T.G., Drenowatz C., McMahon D., Shook R.P., Blair S.N. (2016). Anti-inflammatory Dietary Inflammatory Index scores are associated with healthier scores on other dietary indices. Nutr. Res..

[57] Wu L., Shi Y., Kong C., Zhang J., Chen S. (2022). Dietary Inflammatory Index and Its Association with the Prevalence of Coronary Heart Disease among 45,306 US Adults. Nutrients.

[58] Zare M.J., Ahmadi A., Dehbozorgi S., Zare M., Hejazi N. (2025). The Association Between Children’s Dietary Inflammatory Index (C-DII) and Nutrient Adequacy with Gastrointestinal Symptoms, Sleep Habits, and Autistic Traits. J. Autism Dev. Disord..

[59] Werneck A.O., Araujo R.H.O., Silva D.R., Vancampfort D. (2023). Handgrip strength, physical activity and incident mild cognitive impairment and dementia. Maturitas.

[60] Denison H.J., Jameson K.A., Sayer A.A., Patel H.P., Edwards M.H., Arora T., Dennison E.M., Cooper C., Baird J. (2021). Poor sleep quality and physical performance in older adults. Sleep Health.

[61] Jamal A., Agaki I.T., O’Connor E., King B.A., Kenemer J.B., Neff L. (2014). Current Cigarette Smoking Among Adults—United States, 2005–2013. Morb. Mortal. Wkly. Rep..

[62] Food and Nutrition Service US Department of Agriculture. Average Healthy Eating Index-2010 Scores for Americans by Age Group, WWEIA-NHANES 2011–2012. https://www.fns.usda.gov/sites/default/files/media/file/HEI2010_Age_Groups_2011_2012.pdf.

[63] National Research Council (1989). Recommended Dietary Allowances. Chapter: Protein and Amino Acids.

[64] Carbone J.W., Pasiakos S.M. (2019). Dietary Protein and Muscle Mass: Translating Science to Application and Health Benefit. Nutrients.

[65] Wang Y.C., Bohannon R.W., Li X., Sindhu B., Kapellusch J. (2018). Hand-Grip Strength: Normative Reference Values and Equations for Individuals 18 to 85 Years of Age Residing in the United States. J. Orthop. Sports Phys. Ther..

[66] Bohannon R.W. (2019). Grip Strength: An Indispensable Biomarker for Older Adults. Clin. Interv. Aging.

[67] Pan P.J., Hsu N.W., Lee M.J., Lin Y.Y., Tsai C.C., Lin W.S. (2022). Physical fitness and its correlation with handgrip strength in active community-dwelling older adults. Sci. Rep..

[68] Almashaqbeh S.F., Al-Momani S., Khader A., Qananwah Q., Marabeh S., Maabreh R., Al Badarneh A., Abdullah K. (2022). The Effect of Gender and Arm Anatomical Position on the Hand Grip Strength and Fatigue Resistance during Sustained Maximal Handgrip Effort. J. Biomed. Phys. Eng..

[69] Huemer M.-T., Kluttig A., Fischer B., Ahrens W., Castell S., Ebert N., Gastell S., Jöckel K.-H., Kaaks R., Karch A. (2023). Grip strength values and cut-off points based on over 200,000 adults of the German National Cohort—A comparison to the EWGSOP2 cut-off points. Age Ageing.

[70] Ribeiro L.W., Berndt S., Mielke G.I., Doust J., Mishra G.D. (2024). Factors associated with handgrip strength across the life course: A systematic review. J. Cachexia Sarcopenia Muscle.

[71] Cho E., Soh H.S., Lee J.R., Yun J., Bae W.K., Lee H. (2023). Association between smoking status and handgrip strength in Korean male adults: Based on Korea National Health and Nutrition Examination Survey 2016–2019. Front. Med..

[72] Jackson C.L., Patel S.R., Jackson W.B., Lutsey P.L., Redline S. (2018). Agreement between self-reported and objectively measured sleep duration among white, black, Hispanic, and Chinese adults in the United States: Multi-Ethnic Study of Atherosclerosis. Sleep.

[73] Jackowska M., Ronaldson A., Brown J., Steptoe A. (2016). Biological and psychological correlates of self-reported and objective sleep measures. J. Psychosom. Res..

[74] Howes E.M., Parker M.K., Misyak S.A., DiFeliceantonio A.G., Davy B.M., Brown L.E.C., Hedrick V.E. (2024). The Impact of Weight Bias and Stigma on the 24 h Dietary Recall Process in Adults with Overweight and Obesity: A Pilot Study. Nutrients.

[75] Dyrstad S.M., Hansen B.H., Holme I.M., Anderssen S.A. (2014). Comparison of self-reported versus accelerometer-measured physical activity. Med. Sci. Sports Exerc..

