# Supplementary material for: Protein Intake and Diet Quality Mediate the Relationship Between Sleep and Handgrip Strength in Adults in the HANDLS Study
Source: Nutrients. 2025 May 31;17(11):1900. doi: 10.3390/nu17111900 (PMC12157243; doi:10.3390/nu17111900)
Supplement: Supplementary file 1 [file nutrients-17-01900-s001.zip › nutrients-3598527-supplementary.pdf]

Protein intake and diet quality mediate the relationship between sleep and handgrip strength in adults in the HANDLS study

Supplementary Tables

Table S1. Summary of Pittsburgh Sleep Quality Index responses to component 5

Question	0 times in last month	<1 time/week	1-2 times/week	3+ times/week
Cannot get to sleep within 30 minutes	51.1	3.5	6.7	38.7
Wake up in the middle of the night or early morning	23.2	6.7	14.1	56.0
Have to get up to use the bathroom	27.7	5.9	15.8	50.6
Cannot breathe comfortably	77.1	4.1	8.7	10.0
Cough or snore loudly	70.3	4.1	9.2	16.5
Feel too cold	92.1	2.8	3.4	1.7
Feel too hot	73.4	7.9	12.4	6.3
Had bad dreams	69.4	14.8	11.0	4.8
Have pain	45.1	5.8	18.3	30.8

Table S2. Output for Process macro, Model #4 with sleep quality and handgrip strength incorporating energy-adjusted Dietary Inflammatory Index (e-DII), physical activity, and covariates

HG/BMI (outcome)	β	SE	p
Constant	0.961	0.118	<0.0001
PSQI	-0.007	0.002	0.005
eDII	-0.002	0.009	0.824
Physical activity	-0.037	0.019	0.051
Race	-0.005	0.021	0.815
Sex	0.601	0.021	<0.0001
Poverty	-0.030	0.022	0.164
Age	-0.006	0.001	<0.0001
Smoker	0.097	0.022	<0.0001
Drug user	-0.008	0.030	0.777
Medical condition	-0.165	0.023	<0.0001

R^2 =0.49

Table S3. Output for Process macro, Model #4 with sleep quality and handgrip strength incorporating Healthy Eating Index-2010 (HEI) and physical activity

HG/BMI (outcome)	β	SE	p
Constant	0.848	0.126	<0.0001
PSQI	-0.006	0.002	0.009
HEI	0.002	0.001	0.038
Physical activity	-0.031	0.019	0.107
Race	-0.007	0.021	0.733
Sex	0.605	0.021	<0.0001
Poverty	-0.028	0.022	0.202

Age	-0.006	0.001	<0.0001
Energy	0.000	0.000	0.071
Smoker	0.107	0.022	<0.0001
Drug user	-0.014	0.030	0.648
Medical condition	-0.166	0.024	<0.0001

R^2 =0.49 (p<0.0001)

Table S4. Output for Process macro, Model #4 with sleep quality and handgrip strength incorporating protein intake(g)/kg body weight (Protein/BW) and physical activity

HG/BMI (outcome)	β	SE	p
Constant	0.899	0.116	<0.0001
PSQI	-0.005	0.002	0.034
Protein/BW	0.275	0.030	<0.0001
Physical activity	-0.027	0.018	0.144
Race	-0.016	0.020	0.418
Sex	0.589	0.021	<0.0001
Poverty	-0.044	0.021	0.038
Age	-0.007	0.001	<0.0001
Energy	0.0001	0.000	<0.0001
Smoker	0.074	0.022	<0.001
Drug user	-0.001	0.029	0.973
Medical condition	-0.138	0.023	<0.0001

R^2 =0.53 (p<0.0001)

Table S5. Output for Process macro, Model #4 with sleep duration and handgrip strength incorporating energy-adjusted Dietary Inflammatory Index (e-DII) and physical activity

HG/BMI (outcome)	β	SE	p
Constant	0.874	0.126	<0.0001
Sleep duration	0.009	0.006	0.178
e-DII	-0.004	0.009	0.664
Physical activity	-0.043	0.019	0.025
Race	0.002	0.021	0.930
Sex	0.608	0.021	<0.0001
Poverty	-0.031	0.022	0.154
Age	-0.006	0.001	<0.0001
Smoker	0.090	0.222	0.0001
Drug user	-0.005	0.030	0.866
Medical condition	-0.171	0.024	<0.0001

R^2 =0.49 (p<0.0001)

Table S6. Output for Process macro, Model #4 with sleep duration and handgrip strength incorporating Healthy Eating Index-2010 (HEI) and physical activity

HG/BMI (outcome)	β	SE	p
Constant	0.756	0.132	<0.0001
Sleep duration	0.008	0.006	0.228

HEI	0.002	0.001	0.026
Physical activity	-0.036	0.019	0.060
Race	-0.001	0.021	0.949
Sex	0.612	0.021	<0.0001
Poverty	-0.029	0.022	0.187
Age	-0.006	0.001	<0.0001
Smoker	0.100	0.023	<0.0001
Drug user	-0.011	0.030	0.718
Medical condition	-0.172	0.024	<0.0001

Table S7. Output for Process macro, Model #4 with sleep duration and handgrip strength incorporating protein intake(g)/kg body weight (Protein/BW) and physical activity

**HG/BMI (outcome)**	**β**	**SE**	**p**
Constant	0.848	0.124	<0.0001
Sleep duration	0.003	0.006	0.604
Protein/BW	0.278	0.030	<0.0001
Physical activity	-0.032	0.018	0.079
Race	-0.012	0.020	0.548
Sex	0.592	0.021	<0.0001
Poverty	-0.045	0.021	0.033
Age	-0.007	0.001	<0.0001
Energy	-0.0001	0.0000	<0.0001
Smoker	0.068	0.021	0.002
Drug user	0.002	0.029	0.949
Medical condition	-0.143	0.023	<0.0001

R^2 =0.53 (p<0.0001)
